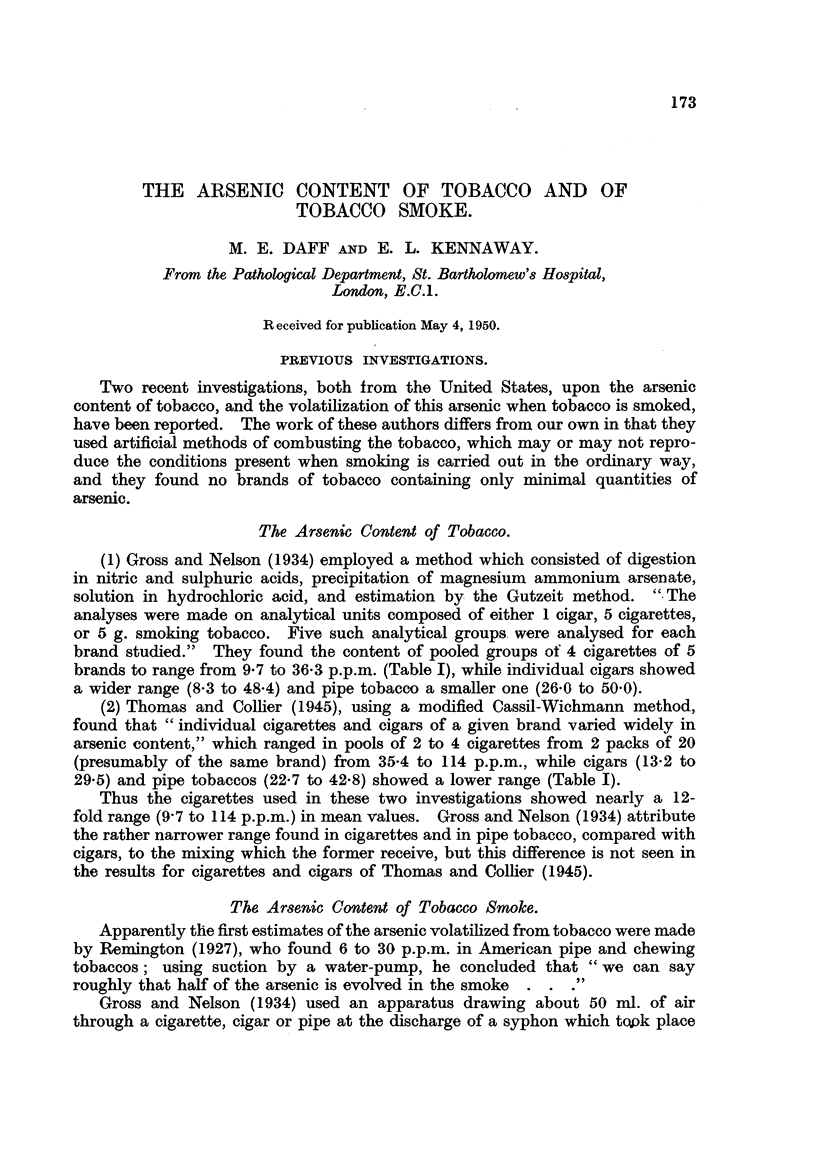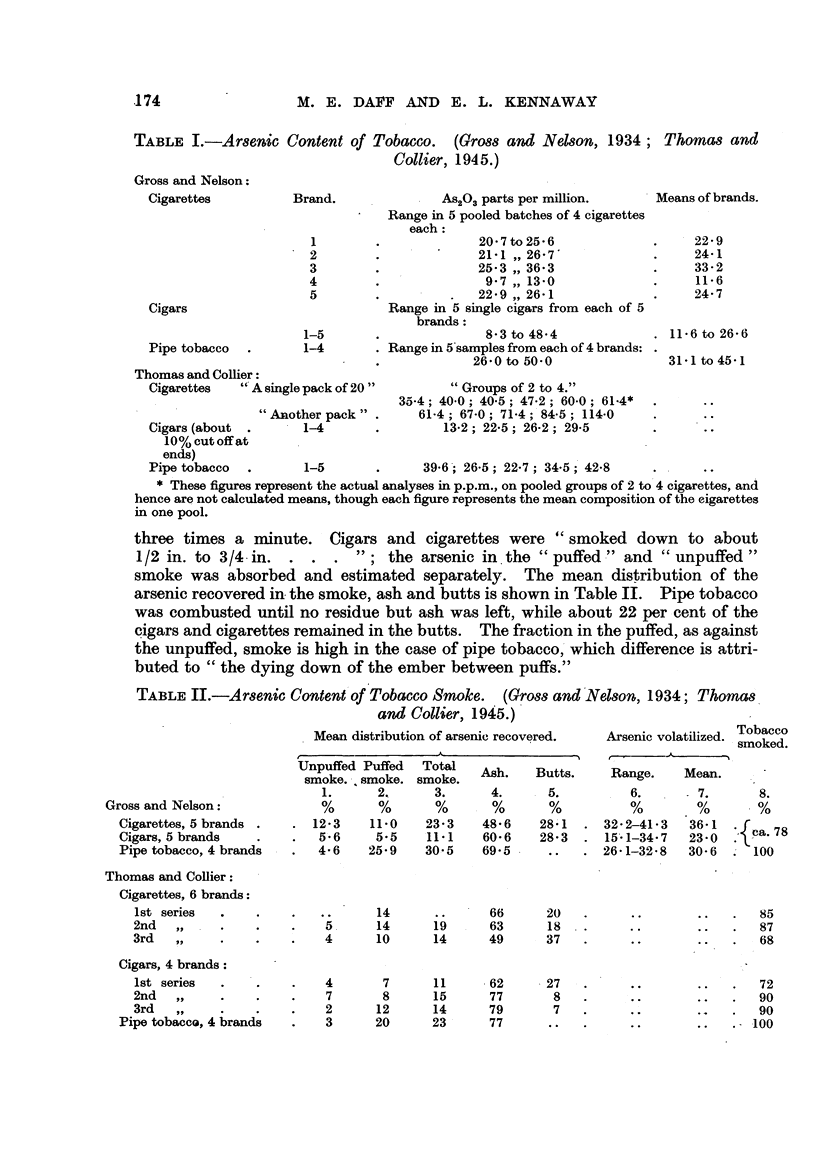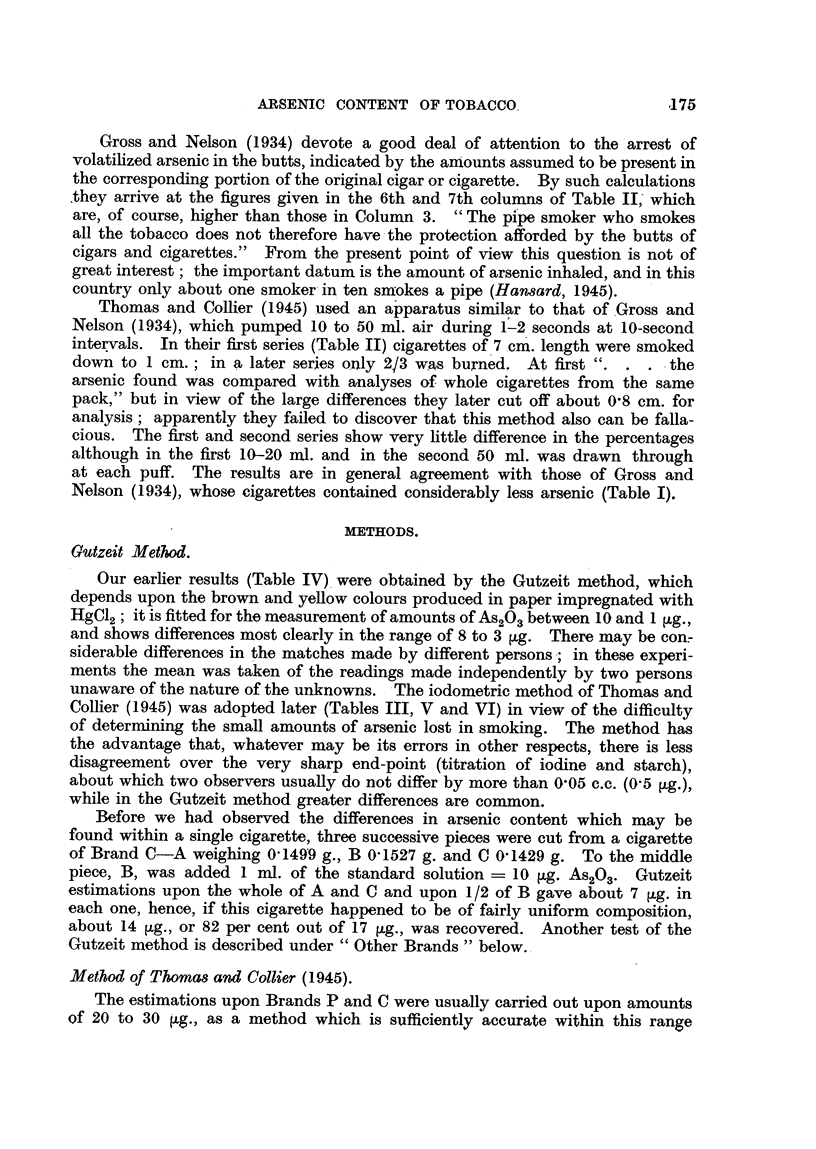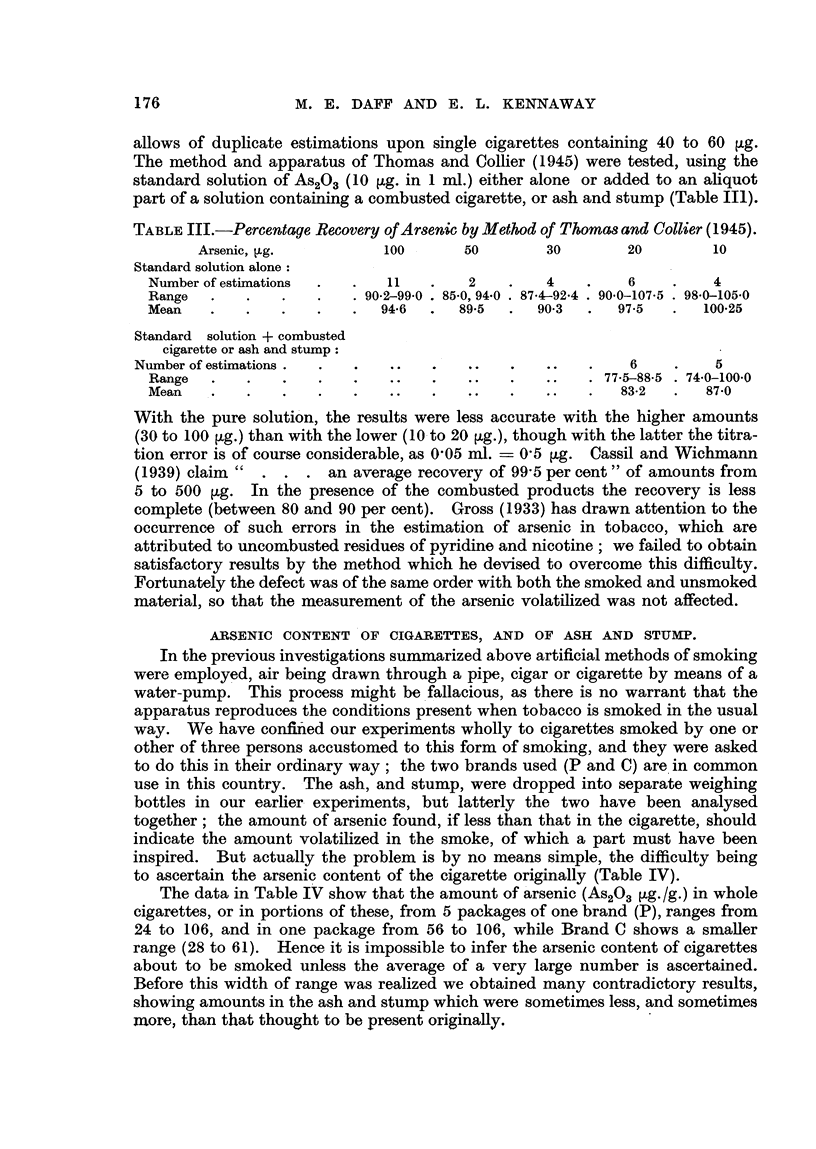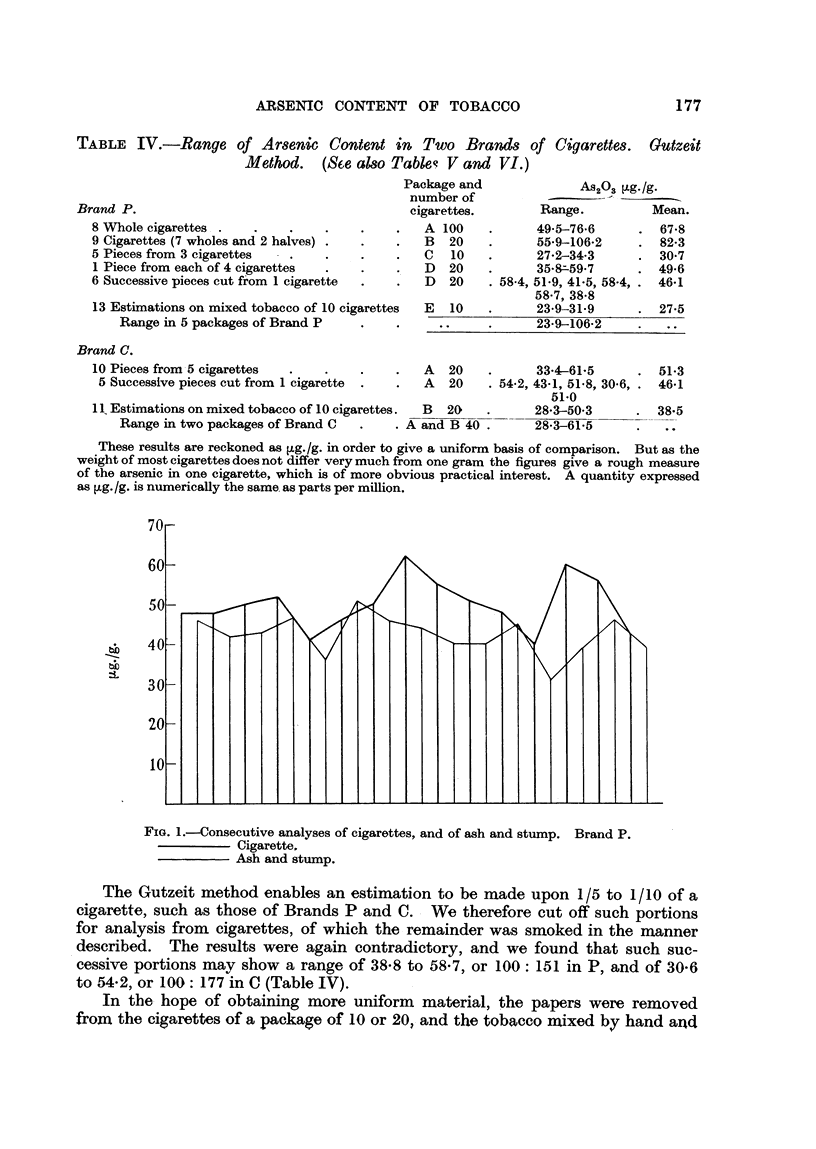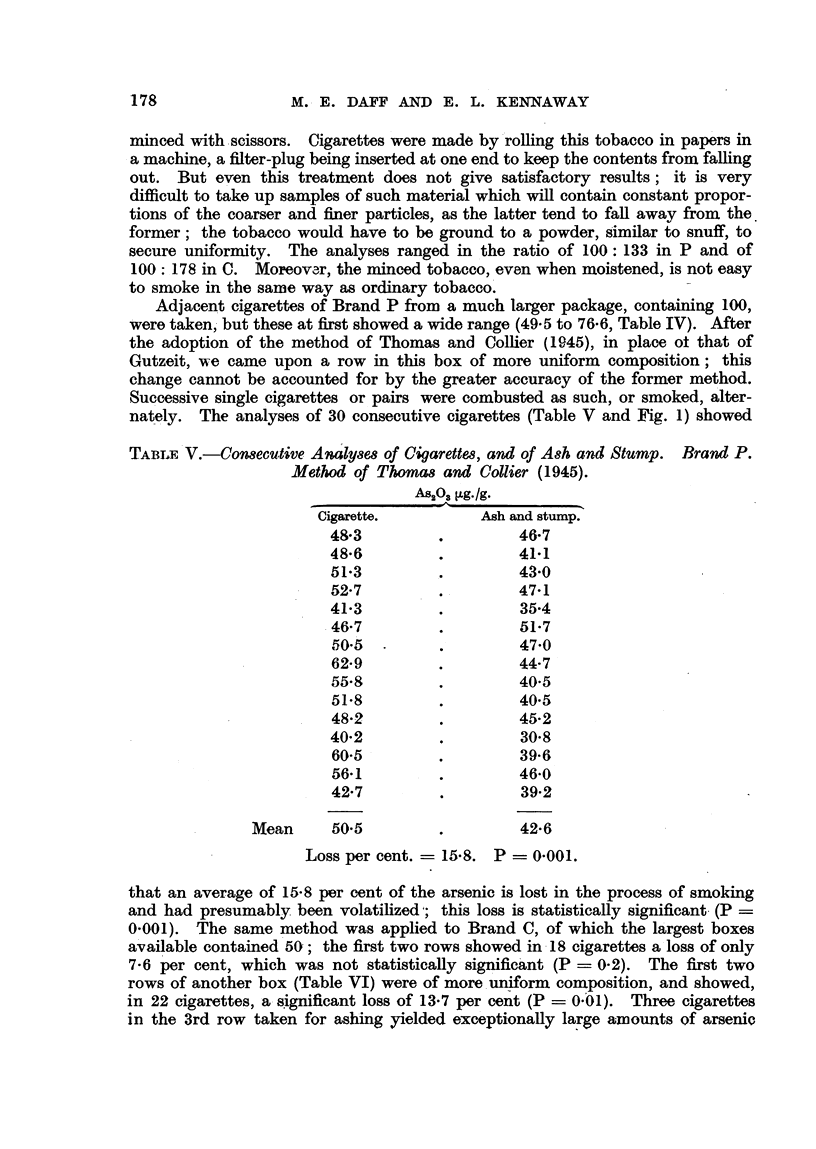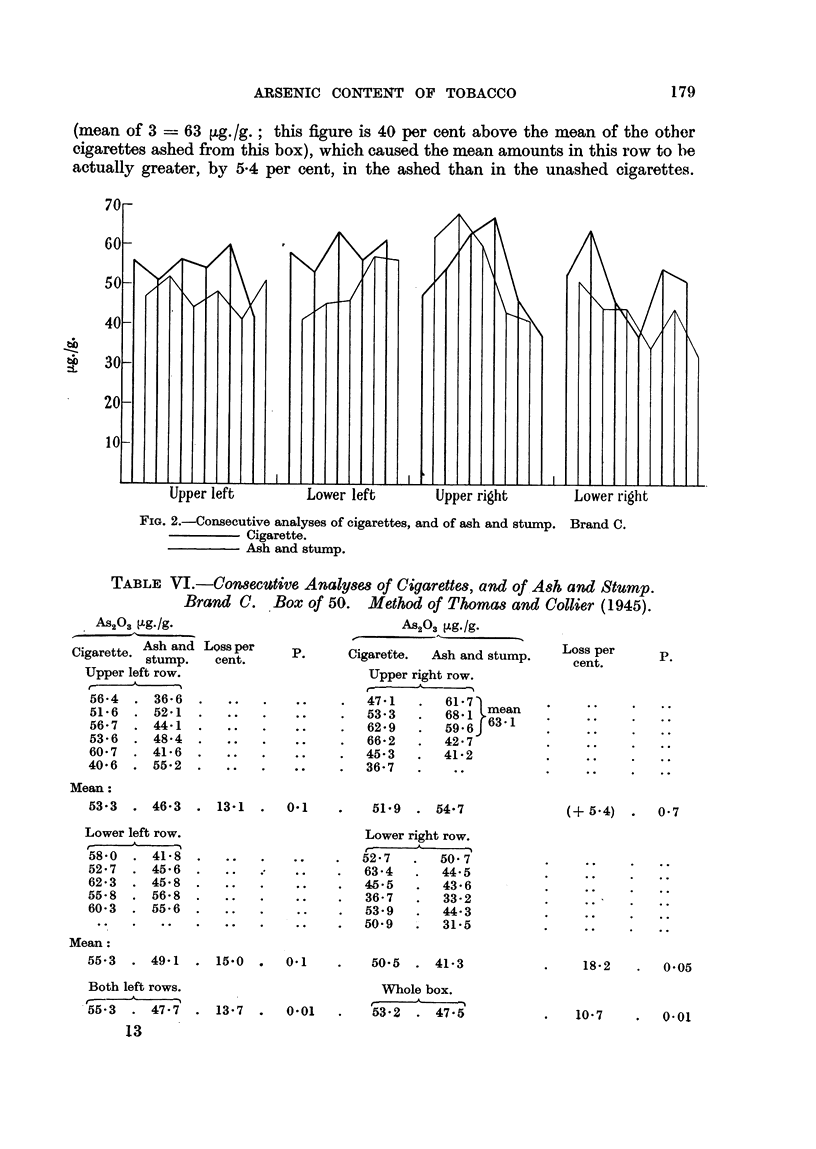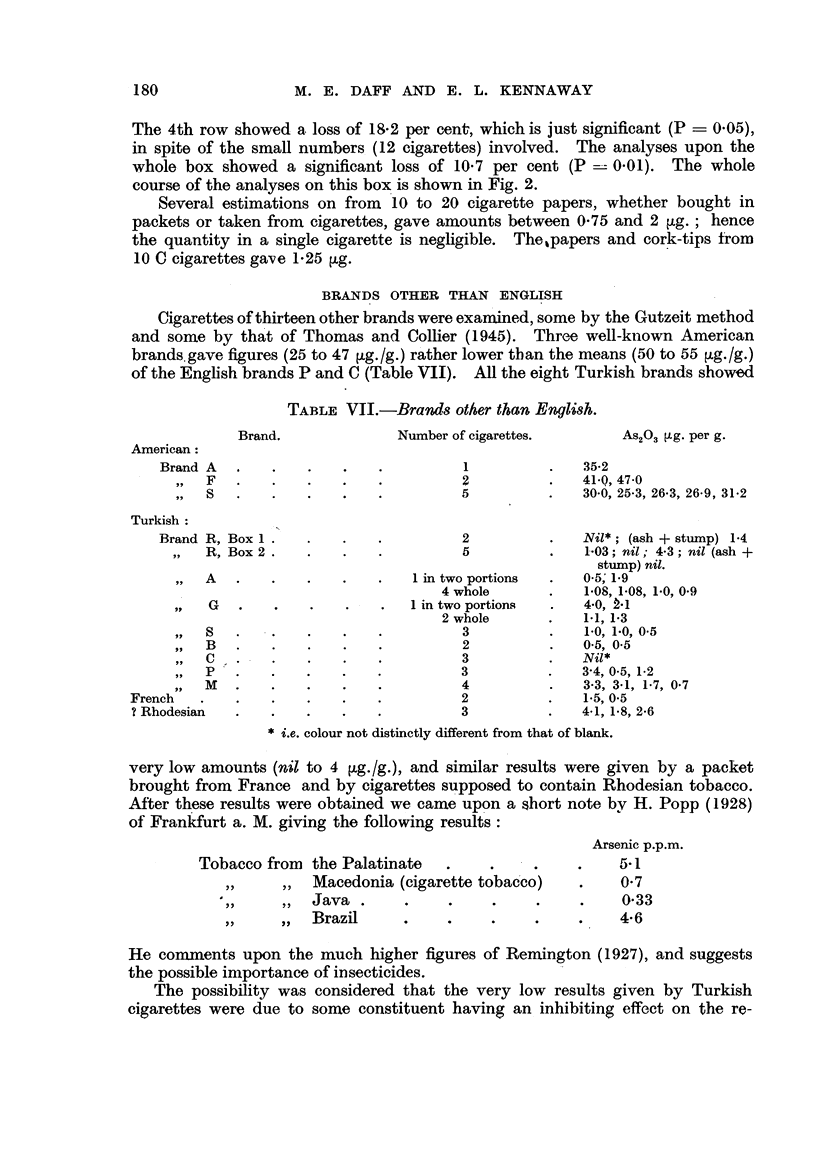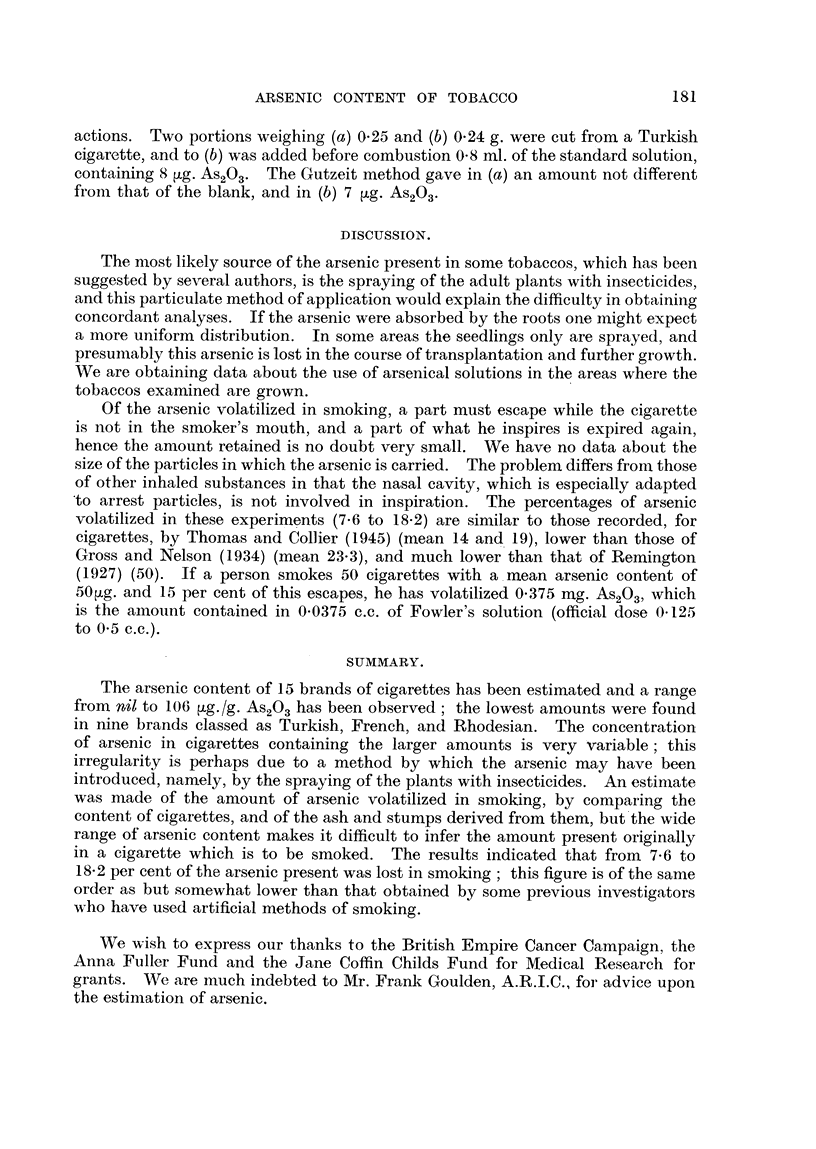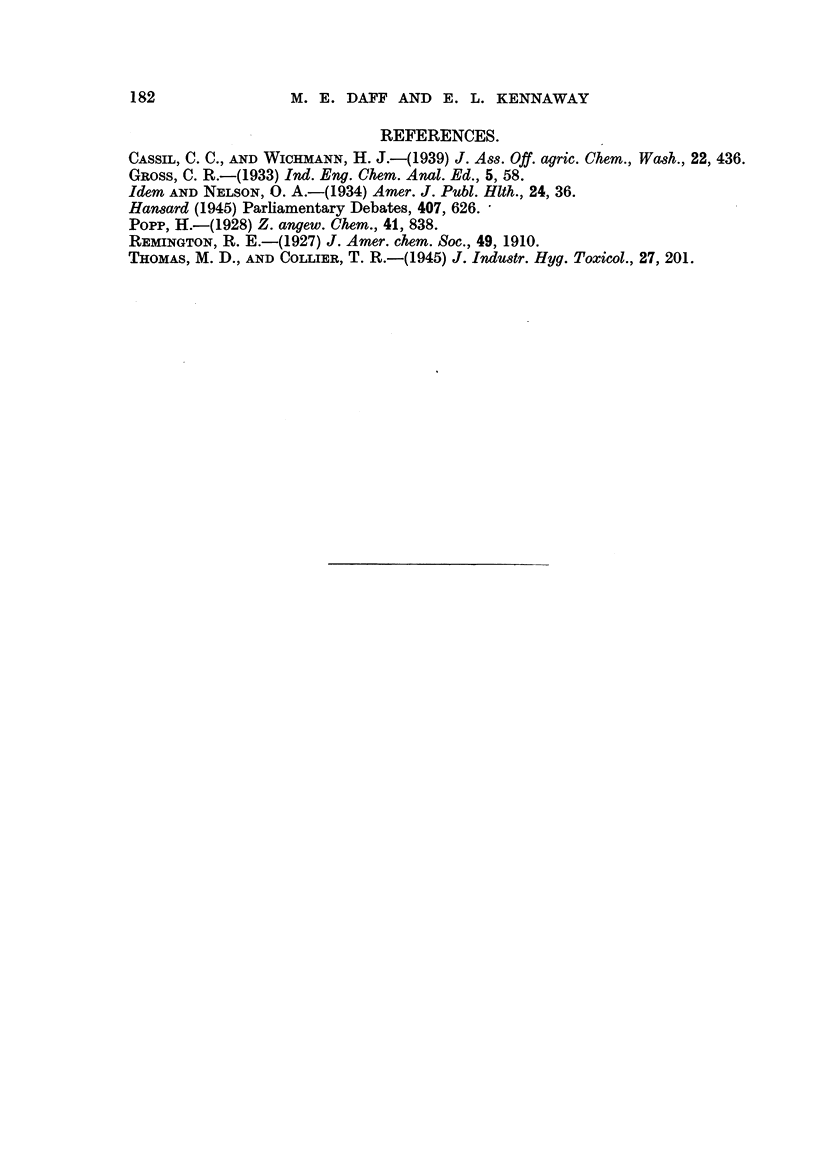# The Arsenic Content of Tobacco and of Tobacco Smoke

**DOI:** 10.1038/bjc.1950.17

**Published:** 1950-06

**Authors:** M. E. Daff, E. L. Kennaway


### THE ARSENIC CONTENT OF TOBACCO AND OF TOBACCO SMOKE.

M. E. DAFF AND E. L. KENNAWAY.

*From the Pathological Department, St. Bartholomew's Hospital, London, E.C.*1.

Received for publication May 4, 1950.

### PREVIOUS INVESTIGATIONS.

Two recent investigations, both from the United States, upon the arsenic content of tobacco, and the volatilization of this arsenic when tobacco is smoked, have been reported. The work of these authors differs from our own in that they used artificial methods of combusting the tobacco, which may or may not reproduce the conditions present when smoking is carried out in the ordinary way, and they found no brands of tobacco containing only minimal quantities of arsenic.

### *The Arsenic Content of Tobacco.*

(1) Gross and Nelson (1934) employed a method which consisted of digestion in nitric and sulphuric acids, precipitation of magnesium ammonium arsenate, solution in hydrochloric acid, and estimation by the Gutzeit method. "The analyses were made on analytical units composed of either 1 cigar, 5 cigarettes, or 5 g. smoking tobacco. Five such analytical groups were analysed for each brand studied." They found the content of pooled groups of 4 cigarettes of 5 brands to range from 9·7 to 36·3 p.p.m. (Table I), while individual cigars showed a wider range (8·3 to 48·4) and pipe tobacco a smaller one (26·0 to 50·0).

(2) Thomas and Collier (1945), using a modified Cassil-Wichmann method, found that "individual cigarettes and cigars of a given brand varied widely in arsenic content," which ranged in pools of 2 to 4 cigarettes from 2 packs of 20 (presumably of the same brand) from 35·4 to 114 p.p.m., while cigars (13·2 to 29·5) and pipe tobaccos (22·7 to 42·8) showed a lower range (Table I).

Thus the cigarettes used in these two investigations showed nearly a 12-fold range (9·7 to 114 p.p.m.) in mean values. Gross and Nelson (1934) attribute the rather narrower range found in cigarettes and in pipe tobacco, compared with cigars, to the mixing which the former receive, but this difference is not seen in the results for cigarettes and cigars of Thomas and Collier (1945).

### *The Arsenic Content of Tobacco Smoke.*

Apparently the first estimates of the arsenic volatilized from tobacco were made by Remington (1927), who found 6 to 30 p.p.m. in American pipe and chewing tobaccos; using suction by a water-pump, he concluded that "we can say roughly that half of the arsenic is evolved in the smoke . . ."

Gross and Nelson (1934) used an apparatus drawing about 50 ml. of air through a cigarette, cigar or pipe at the discharge of a syphon which took place

TABLE I.—*Arsenic Content of Tobacco.* (*Gross and Nelson,* 1934 ; *Thomas and Collier,* 1945.)

| | Brand. | $As_2O_3$ parts per million. | Means of brands. |
|---|---|---|---|
| Gross and Nelson: | | | |
| Cigarettes | | Range in 5 pooled batches of 4 cigarettes each : | |
| | 1 | 20·7 to 25·6 | 22·9 |
| | 2 | 21·1 „ 26·7 | 24·1 |
| | 3 | 25·3 „ 36·3 | 33·2 |
| | 4 | 9·7 „ 13·0 | 11·6 |
| | 5 | 22·9 „ 26·1 | 24·7 |
| Cigars | | Range in 5 single cigars from each of 5 brands : | |
| | 1–5 | 8·3 to 48·4 | 11·6 to 26·6 |
| Pipe tobacco | 1–4 | Range in 5 samples from each of 4 brands: 26·0 to 50·0 | 31·1 to 45·1 |
| Thomas and Collier: | | | |
| Cigarettes | " A single pack of 20 " | " Groups of 2 to 4." 35·4 ; 40·0 ; 40·5 ; 47·2 ; 60·0 ; 61·4* | .. |
| | " Another pack " | 61·4 ; 67·0 ; 71·4 ; 84·5 ; 114·0 | .. |
| Cigars (about 10% cut off at ends) | 1–4 | 13·2 ; 22·5 ; 26·2 ; 29·5 | .. |
| Pipe tobacco | 1–5 | 39·6 ; 26·5 ; 22·7 ; 34·5 ; 42·8 | .. |

* These figures represent the actual analyses in p.p.m., on pooled groups of 2 to 4 cigarettes, and hence are not calculated means, though each figure represents the mean composition of the cigarettes in one pool.

three times a minute. Cigars and cigarettes were " smoked down to about 1/2 in. to 3/4 in. . . . " ; the arsenic in the " puffed " and " unpuffed " smoke was absorbed and estimated separately. The mean distribution of the arsenic recovered in the smoke, ash and butts is shown in Table II. Pipe tobacco was combusted until no residue but ash was left, while about 22 per cent of the cigars and cigarettes remained in the butts. The fraction in the puffed, as against the unpuffed, smoke is high in the case of pipe tobacco, which difference is attributed to " the dying down of the ember between puffs."

TABLE II.—*Arsenic Content of Tobacco Smoke.* (*Gross and Nelson,* 1934; *Thomas and Collier,* 1945.)

| | Mean distribution of arsenic recovered. | | | | | Arsenic volatilized. | | Tobacco smoked. |
|---|---|---|---|---|---|---|---|---|
| | Unpuffed smoke. 1. | Puffed smoke. 2. | Total smoke. 3. | Ash. 4. | Butts. 5. | Range. 6. | Mean. 7. | 8. |
| Gross and Nelson: | % | % | % | % | % | % | % | % |
| Cigarettes, 5 brands | 12·3 | 11·0 | 23·3 | 48·6 | 28·1 | 32·2–41·3 | 36·1 | ca. 78 (Cigarettes and Cigars) |
| Cigars, 5 brands | 5·6 | 5·5 | 11·1 | 60·6 | 28·3 | 15·1–34·7 | 23·0 | |
| Pipe tobacco, 4 brands | 4·6 | 25·9 | 30·5 | 69·5 | .. | 26·1–32·8 | 30·6 | 100 |
| Thomas and Collier: | | | | | | | | |
| Cigarettes, 6 brands: | | | | | | | | |
| 1st series | .. | 14 | .. | 66 | 20 | .. | .. | 85 |
| 2nd „ | 5 | 14 | 19 | 63 | 18 | .. | .. | 87 |
| 3rd „ | 4 | 10 | 14 | 49 | 37 | .. | .. | 68 |
| Cigars, 4 brands: | | | | | | | | |
| 1st series | 4 | 7 | 11 | 62 | 27 | .. | .. | 72 |
| 2nd „ | 7 | 8 | 15 | 77 | 8 | .. | .. | 90 |
| 3rd „ | 2 | 12 | 14 | 79 | 7 | .. | .. | 90 |
| Pipe tobacco, 4 brands | 3 | 20 | 23 | 77 | .. | .. | .. | 100 |

Gross and Nelson (1934) devote a good deal of attention to the arrest of volatilized arsenic in the butts, indicated by the amounts assumed to be present in the corresponding portion of the original cigar or cigarette. By such calculations they arrive at the figures given in the 6th and 7th columns of Table II, which are, of course, higher than those in Column 3. "The pipe smoker who smokes all the tobacco does not therefore have the protection afforded by the butts of cigars and cigarettes." From the present point of view this question is not of great interest; the important datum is the amount of arsenic inhaled, and in this country only about one smoker in ten smokes a pipe (*Hansard*, 1945).

Thomas and Collier (1945) used an apparatus similar to that of Gross and Nelson (1934), which pumped 10 to 50 ml. air during 1–2 seconds at 10-second intervals. In their first series (Table II) cigarettes of 7 cm. length were smoked down to 1 cm.; in a later series only 2/3 was burned. At first ". . . the arsenic found was compared with analyses of whole cigarettes from the same pack," but in view of the large differences they later cut off about 0·8 cm. for analysis; apparently they failed to discover that this method also can be fallacious. The first and second series show very little difference in the percentages although in the first 10–20 ml. and in the second 50 ml. was drawn through at each puff. The results are in general agreement with those of Gross and Nelson (1934), whose cigarettes contained considerably less arsenic (Table I).

### METHODS.

### *Gutzeit Method.*

Our earlier results (Table IV) were obtained by the Gutzeit method, which depends upon the brown and yellow colours produced in paper impregnated with $HgCl_2$; it is fitted for the measurement of amounts of $As_2O_3$ between 10 and 1 $\mu$g., and shows differences most clearly in the range of 8 to 3 $\mu$g. There may be considerable differences in the matches made by different persons; in these experiments the mean was taken of the readings made independently by two persons unaware of the nature of the unknowns. The iodometric method of Thomas and Collier (1945) was adopted later (Tables III, V and VI) in view of the difficulty of determining the small amounts of arsenic lost in smoking. The method has the advantage that, whatever may be its errors in other respects, there is less disagreement over the very sharp end-point (titration of iodine and starch), about which two observers usually do not differ by more than 0·05 c.c. (0·5 $\mu$g.), while in the Gutzeit method greater differences are common.

Before we had observed the differences in arsenic content which may be found within a single cigarette, three successive pieces were cut from a cigarette of Brand C—A weighing 0·1499 g., B 0·1527 g. and C 0·1429 g. To the middle piece, B, was added 1 ml. of the standard solution = 10 $\mu$g. $As_2O_3$. Gutzeit estimations upon the whole of A and C and upon 1/2 of B gave about 7 $\mu$g. in each one, hence, if this cigarette happened to be of fairly uniform composition, about 14 $\mu$g., or 82 per cent out of 17 $\mu$g., was recovered. Another test of the Gutzeit method is described under "Other Brands" below.

### *Method of Thomas and Collier* (1945).

The estimations upon Brands P and C were usually carried out upon amounts of 20 to 30 $\mu$g., as a method which is sufficiently accurate within this range allows of duplicate estimations upon single cigarettes containing 40 to 60 μg. The method and apparatus of Thomas and Collier (1945) were tested, using the standard solution of $As_2O_3$ (10 μg. in 1 ml.) either alone or added to an aliquot part of a solution containing a combusted cigarette, or ash and stump (Table III).

TABLE III.—*Percentage Recovery of Arsenic by Method of Thomas and Collier* (1945).

| Arsenic, μg. | 100 | 50 | 30 | 20 | 10 |
|---|---|---|---|---|---|
| Standard solution alone : | | | | | |
| Number of estimations | 11 | 2 | 4 | 6 | 4 |
| Range | 90·2–99·0 | 85·0, 94·0 | 87·4–92·4 | 90·0–107·5 | 98·0–105·0 |
| Mean | 94·6 | 89·5 | 90·3 | 97·5 | 100·25 |
| Standard solution + combusted cigarette or ash and stump : | | | | | |
| Number of estimations | .. | .. | .. | 6 | 5 |
| Range | .. | .. | .. | 77·5–88·5 | 74·0–100·0 |
| Mean | .. | .. | .. | 83·2 | 87·0 |

With the pure solution, the results were less accurate with the higher amounts (30 to 100 μg.) than with the lower (10 to 20 μg.), though with the latter the titration error is of course considerable, as 0·05 ml. = 0·5 μg. Cassil and Wichmann (1939) claim " . . . an average recovery of 99·5 per cent" of amounts from 5 to 500 μg. In the presence of the combusted products the recovery is less complete (between 80 and 90 per cent). Gross (1933) has drawn attention to the occurrence of such errors in the estimation of arsenic in tobacco, which are attributed to uncombusted residues of pyridine and nicotine ; we failed to obtain satisfactory results by the method which he devised to overcome this difficulty. Fortunately the defect was of the same order with both the smoked and unsmoked material, so that the measurement of the arsenic volatilized was not affected.

### ARSENIC CONTENT OF CIGARETTES, AND OF ASH AND STUMP.

In the previous investigations summarized above artificial methods of smoking were employed, air being drawn through a pipe, cigar or cigarette by means of a water-pump. This process might be fallacious, as there is no warrant that the apparatus reproduces the conditions present when tobacco is smoked in the usual way. We have confined our experiments wholly to cigarettes smoked by one or other of three persons accustomed to this form of smoking, and they were asked to do this in their ordinary way ; the two brands used (P and C) are in common use in this country. The ash, and stump, were dropped into separate weighing bottles in our earlier experiments, but latterly the two have been analysed together ; the amount of arsenic found, if less than that in the cigarette, should indicate the amount volatilized in the smoke, of which a part must have been inspired. But actually the problem is by no means simple, the difficulty being to ascertain the arsenic content of the cigarette originally (Table IV).

The data in Table IV show that the amount of arsenic ($As_2O_3$ μg./g.) in whole cigarettes, or in portions of these, from 5 packages of one brand (P), ranges from 24 to 106, and in one package from 56 to 106, while Brand C shows a smaller range (28 to 61). Hence it is impossible to infer the arsenic content of cigarettes about to be smoked unless the average of a very large number is ascertained. Before this width of range was realized we obtained many contradictory results, showing amounts in the ash and stump which were sometimes less, and sometimes more, than that thought to be present originally.

TABLE IV.—*Range of Arsenic Content in Two Brands of Cigarettes. Gutzeit Method.* (*See also Tables V and VI.*)

| | Package and number of cigarettes. | $As_2O_3$ µg./g. Range. | Mean. |
|---|---|---|---|
| *Brand P.* | | | |
| 8 Whole cigarettes | A 100 | 49·5–76·6 | 67·8 |
| 9 Cigarettes (7 wholes and 2 halves) | B 20 | 55·9–106·2 | 82·3 |
| 5 Pieces from 3 cigarettes | C 10 | 27·2–34·3 | 30·7 |
| 1 Piece from each of 4 cigarettes | D 20 | 35·8–59·7 | 49·6 |
| 6 Successive pieces cut from 1 cigarette | D 20 | 58·4, 51·9, 41·5, 58·4, 58·7, 38·8 | 46·1 |
| 13 Estimations on mixed tobacco of 10 cigarettes | E 10 | 23·9–31·9 | 27·5 |
| Range in 5 packages of Brand P | .. | 23·9–106·2 | .. |
| *Brand C.* | | | |
| 10 Pieces from 5 cigarettes | A 20 | 33·4–61·5 | 51·3 |
| 5 Successive pieces cut from 1 cigarette | A 20 | 54·2, 43·1, 51·8, 30·6, 51·0 | 46·1 |
| 11 Estimations on mixed tobacco of 10 cigarettes | B 20 | 28·3–50·3 | 38·5 |
| Range in two packages of Brand C | A and B 40 | 28·3–61·5 | .. |

These results are reckoned as µg./g. in order to give a uniform basis of comparison. But as the weight of most cigarettes does not differ very much from one gram the figures give a rough measure of the arsenic in one cigarette, which is of more obvious practical interest. A quantity expressed as µg./g. is numerically the same as parts per million.

FIG. 1.—Consecutive analyses of cigarettes, and of ash and stump. Brand P.
——————— Cigarette.
——————— Ash and stump.

The Gutzeit method enables an estimation to be made upon 1/5 to 1/10 of a cigarette, such as those of Brands P and C. We therefore cut off such portions for analysis from cigarettes, of which the remainder was smoked in the manner described. The results were again contradictory, and we found that such successive portions may show a range of 38·8 to 58·7, or 100 : 151 in P, and of 30·6 to 54·2, or 100 : 177 in C (Table IV).

In the hope of obtaining more uniform material, the papers were removed from the cigarettes of a package of 10 or 20, and the tobacco mixed by hand and minced with scissors. Cigarettes were made by rolling this tobacco in papers in a machine, a filter-plug being inserted at one end to keep the contents from falling out. But even this treatment does not give satisfactory results; it is very difficult to take up samples of such material which will contain constant proportions of the coarser and finer particles, as the latter tend to fall away from the former; the tobacco would have to be ground to a powder, similar to snuff, to secure uniformity. The analyses ranged in the ratio of 100 : 133 in P and of 100 : 178 in C. Moreover, the minced tobacco, even when moistened, is not easy to smoke in the same way as ordinary tobacco.

Adjacent cigarettes of Brand P from a much larger package, containing 100, were taken, but these at first showed a wide range (49·5 to 76·6, Table IV). After the adoption of the method of Thomas and Collier (1945), in place of that of Gutzeit, we came upon a row in this box of more uniform composition; this change cannot be accounted for by the greater accuracy of the former method. Successive single cigarettes or pairs were combusted as such, or smoked, alternately. The analyses of 30 consecutive cigarettes (Table V and Fig. 1) showed

TABLE V.—*Consecutive Analyses of Cigarettes, and of Ash and Stump. Brand P. Method of Thomas and Collier* (1945).

| | $As_2O_3$ µg./g. | |
|---|---|---|
| | Cigarette. | Ash and stump. |
| | 48·3 | 46·7 |
| | 48·6 | 41·1 |
| | 51·3 | 43·0 |
| | 52·7 | 47·1 |
| | 41·3 | 35·4 |
| | 46·7 | 51·7 |
| | 50·5 | 47·0 |
| | 62·9 | 44·7 |
| | 55·8 | 40·5 |
| | 51·8 | 40·5 |
| | 48·2 | 45·2 |
| | 40·2 | 30·8 |
| | 60·5 | 39·6 |
| | 56·1 | 46·0 |
| | 42·7 | 39·2 |
| Mean | 50·5 | 42·6 |

Loss per cent. = 15·8. P = 0·001.

that an average of 15·8 per cent of the arsenic is lost in the process of smoking and had presumably been volatilized; this loss is statistically significant (P = 0·001). The same method was applied to Brand C, of which the largest boxes available contained 50; the first two rows showed in 18 cigarettes a loss of only 7·6 per cent, which was not statistically significant (P = 0·2). The first two rows of another box (Table VI) were of more uniform composition, and showed, in 22 cigarettes, a significant loss of 13·7 per cent (P = 0·01). Three cigarettes in the 3rd row taken for ashing yielded exceptionally large amounts of arsenic

(mean of 3 = 63 μg./g. ; this figure is 40 per cent above the mean of the other cigarettes ashed from this box), which caused the mean amounts in this row to be actually greater, by 5·4 per cent, in the ashed than in the unashed cigarettes.

Fig. 2.—Consecutive analyses of cigarettes, and of ash and stump. Brand C.
——— Cigarette.
——— Ash and stump.

Table VI.—*Consecutive Analyses of Cigarettes, and of Ash and Stump. Brand C. Box of* 50. *Method of Thomas and Collier* (1945).

| $As_2O_3$ μg./g. Cigarette. | Ash and stump. | Loss per cent. | P. | $As_2O_3$ μg./g. Cigarette. | Ash and stump. | Loss per cent. | P. |
|---|---|---|---|---|---|---|---|
| Upper left row. | | | | Upper right row. | | | |
| 56·4 | 36·6 | .. | .. | 47·1 | 61·7 | .. | .. |
| 51·6 | 52·1 | .. | .. | 53·3 | 68·1 } mean 63·1 | .. | .. |
| 56·7 | 44·1 | .. | .. | 62·9 | 59·6 | .. | .. |
| 53·6 | 48·4 | .. | .. | 66·2 | 42·7 | .. | .. |
| 60·7 | 41·6 | .. | .. | 45·3 | 41·2 | .. | .. |
| 40·6 | 55·2 | .. | .. | 36·7 | .. | .. | .. |
| Mean : | | | | | | | |
| 53·3 | 46·3 | 13·1 | 0·1 | 51·9 | 54·7 | (+ 5·4) | 0·7 |
| Lower left row. | | | | Lower right row. | | | |
| 58·0 | 41·8 | .. | .. | 52·7 | 50·7 | .. | .. |
| 52·7 | 45·6 | .. | .. | 63·4 | 44·5 | .. | .. |
| 62·3 | 45·8 | .. | .. | 45·5 | 43·6 | .. | .. |
| 55·8 | 56·8 | .. | .. | 36·7 | 33·2 | .. | .. |
| 60·3 | 55·6 | .. | .. | 53·9 | 44·3 | .. | .. |
| .. | .. | .. | .. | 50·9 | 31·5 | .. | .. |
| Mean : | | | | | | | |
| 55·3 | 49·1 | 15·0 | 0·1 | 50·5 | 41·3 | 18·2 | 0·05 |
| Both left rows. | | | | Whole box. | | | |
| 55·3 | 47·7 | 13·7 | 0·01 | 53·2 | 47·5 | 10·7 | 0·01 |

The 4th row showed a loss of 18·2 per cent, which is just significant ($P = 0{\cdot}05$), in spite of the small numbers (12 cigarettes) involved. The analyses upon the whole box showed a significant loss of 10·7 per cent ($P = 0{\cdot}01$). The whole course of the analyses on this box is shown in Fig. 2.

Several estimations on from 10 to 20 cigarette papers, whether bought in packets or taken from cigarettes, gave amounts between 0·75 and 2 μg.; hence the quantity in a single cigarette is negligible. The papers and cork-tips from 10 C cigarettes gave 1·25 μg.

### BRANDS OTHER THAN ENGLISH

Cigarettes of thirteen other brands were examined, some by the Gutzeit method and some by that of Thomas and Collier (1945). Three well-known American brands gave figures (25 to 47 μg./g.) rather lower than the means (50 to 55 μg./g.) of the English brands P and C (Table VII). All the eight Turkish brands showed

TABLE VII.—*Brands other than English.*

| Brand. | Number of cigarettes. | $As_2O_3$ μg. per g. |
|---|---|---|
| American : | | |
| Brand A | 1 | 35·2 |
| ,, F | 2 | 41·0, 47·0 |
| ,, S | 5 | 30·0, 25·3, 26·3, 26·9, 31·2 |
| Turkish : | | |
| Brand R, Box 1 | 2 | *Nil** ; (ash + stump) 1·4 |
| ,, R, Box 2 | 5 | 1·03 ; *nil* ; 4·3 ; *nil* (ash + stump) *nil.* |
| ,, A | 1 in two portions | 0·5, 1·9 |
| | 4 whole | 1·08, 1·08, 1·0, 0·9 |
| ,, G | 1 in two portions | 4·0, 2·1 |
| | 2 whole | 1·1, 1·3 |
| ,, S | 3 | 1·0, 1·0, 0·5 |
| ,, B | 2 | 0·5, 0·5 |
| ,, C | 3 | *Nil** |
| ,, P | 3 | 3·4, 0·5, 1·2 |
| ,, M | 4 | 3·3, 3·1, 1·7, 0·7 |
| French | 2 | 1·5, 0·5 |
| ? Rhodesian | 3 | 4·1, 1·8, 2·6 |

* *i.e.* colour not distinctly different from that of blank.

very low amounts (*nil* to 4 μg./g.), and similar results were given by a packet brought from France and by cigarettes supposed to contain Rhodesian tobacco. After these results were obtained we came upon a short note by H. Popp (1928) of Frankfurt a. M. giving the following results :

| | Arsenic p.p.m. |
|---|---|
| Tobacco from the Palatinate | 5·1 |
| ,, ,, Macedonia (cigarette tobacco) | 0·7 |
| ,, ,, Java | 0·33 |
| ,, ,, Brazil | 4·6 |

He comments upon the much higher figures of Remington (1927), and suggests the possible importance of insecticides.

The possibility was considered that the very low results given by Turkish cigarettes were due to some constituent having an inhibiting effect on the reactions. Two portions weighing (*a*) 0·25 and (*b*) 0·24 g. were cut from a Turkish cigarette, and to (*b*) was added before combustion 0·8 ml. of the standard solution, containing 8 μg. $As_2O_3$. The Gutzeit method gave in (*a*) an amount not different from that of the blank, and in (*b*) 7 μg. $As_2O_3$.

### DISCUSSION.

The most likely source of the arsenic present in some tobaccos, which has been suggested by several authors, is the spraying of the adult plants with insecticides, and this particulate method of application would explain the difficulty in obtaining concordant analyses. If the arsenic were absorbed by the roots one might expect a more uniform distribution. In some areas the seedlings only are sprayed, and presumably this arsenic is lost in the course of transplantation and further growth. We are obtaining data about the use of arsenical solutions in the areas where the tobaccos examined are grown.

Of the arsenic volatilized in smoking, a part must escape while the cigarette is not in the smoker's mouth, and a part of what he inspires is expired again, hence the amount retained is no doubt very small. We have no data about the size of the particles in which the arsenic is carried. The problem differs from those of other inhaled substances in that the nasal cavity, which is especially adapted to arrest particles, is not involved in inspiration. The percentages of arsenic volatilized in these experiments (7·6 to 18·2) are similar to those recorded, for cigarettes, by Thomas and Collier (1945) (mean 14 and 19), lower than those of Gross and Nelson (1934) (mean 23·3), and much lower than that of Remington (1927) (50). If a person smokes 50 cigarettes with a mean arsenic content of 50μg. and 15 per cent of this escapes, he has volatilized 0·375 mg. $As_2O_3$, which is the amount contained in 0·0375 c.c. of Fowler's solution (official dose 0·125 to 0·5 c.c.).

### SUMMARY.

The arsenic content of 15 brands of cigarettes has been estimated and a range from *nil* to 106 μg./g. $As_2O_3$ has been observed ; the lowest amounts were found in nine brands classed as Turkish, French, and Rhodesian. The concentration of arsenic in cigarettes containing the larger amounts is very variable ; this irregularity is perhaps due to a method by which the arsenic may have been introduced, namely, by the spraying of the plants with insecticides. An estimate was made of the amount of arsenic volatilized in smoking, by comparing the content of cigarettes, and of the ash and stumps derived from them, but the wide range of arsenic content makes it difficult to infer the amount present originally in a cigarette which is to be smoked. The results indicated that from 7·6 to 18·2 per cent of the arsenic present was lost in smoking ; this figure is of the same order as but somewhat lower than that obtained by some previous investigators who have used artificial methods of smoking.

We wish to express our thanks to the British Empire Cancer Campaign, the Anna Fuller Fund and the Jane Coffin Childs Fund for Medical Research for grants. We are much indebted to Mr. Frank Goulden, A.R.I.C., for advice upon the estimation of arsenic.

### REFERENCES.

CASSIL, C. C., AND WICHMANN, H. J.—(1939) *J. Ass. Off. agric. Chem., Wash.*, **22**, 436.
GROSS, C. R.—(1933) *Ind. Eng. Chem. Anal. Ed.*, **5**, 58.
*Idem* AND NELSON, O. A.—(1934) *Amer. J. Publ. Hlth.*, **24**, 36.
*Hansard* (1945) Parliamentary Debates, **407**, 626.
POPP, H.—(1928) *Z. angew. Chem.*, **41**, 838.
REMINGTON, R. E.—(1927) *J. Amer. chem. Soc.*, **49**, 1910.
THOMAS, M. D., AND COLLIER, T. R.—(1945) *J. Industr. Hyg. Toxicol.*, **27**, 201.